# Trivalent Influenza Vaccine Adverse Event Analysis Based On MedDRA System Organ Classes Using VAERS Data

**Published:** 2015

**Authors:** Cui Tao, Jingcheng Du, Yi Cai, Yong Chen

**Affiliations:** a School of Biomedical Informatics, The University of Texas Health Science Center at Houston, TX, USA; b School of Public Health, The University of Texas Health Science Center at Houston, TX, USA

**Keywords:** Influnza vaccine adverse event, MedDRA, VAERS

## Abstract

We studied serious reports following influnza vaccine from VAERS database in year 2011. Our statistical analyses revealed differences of reactions among different age groups and between genders. The results may lead to additional studies to uncover factors contributing to the individual differences in susceptibility to influenza infection.

## Introduction

Seasonal influenza is a common vaccine-preventable disease with substantial morbidity. The social burden of seasonal flu can be substantial [1]. Annual vaccination is the most effective strategy to prevent influenza [2]. While their benefits far overweigh their risks and costs, influenza vaccines are accompanied with specific adverse events. Post-approval surveillance of vaccine adverse events is critically needed to assess the vaccine safety throughout its life on the market. The Vaccine Adverse Event Reporting System (VAERS) is a passive surveillance system to monitor vaccine safety after the administration of vaccines licensed in the United States [3]. In this study, we explored statistical analysis on annotated symptoms in the VAERS reports for patients with different genders and ages.

## Materials and Methods

We searched the VAERS for US reports after Trivalent Influenza Vaccine (FLU3) in year 2011 and extracted serious reports (i.e., death, life-threatening illness, hospitalization, prolonged hospitalization, or permanent disability). For each report, the VAERS provides annotations for post-vaccination symptoms in Medical Dictionary for Regulatory Activities (MedDRA) terms [4]. To faciliate further statistical analysis, we further grouped these symptoms based on the MedDRA System Organ Class (SOC) using the NCBO Web Services [5].

To model the total number of serious symptoms per subject, we fit a zero-truncated Poisson regression on age groups and gender because a subject has to have at least one symptom to be included in the VAERS database. To study the risks of having serious symptom for each SOC type, we use logistic regression on age groups and gender.

## Results

During the study period, VAERS received 7986 FLU3 reports, 638 were serious. Out of the 638 reports, 324 were for female patients, 295 were for male patients, and 134, 156, 110, 185 were for patients in age groups 0.5–17, 17–49, 49–64, and >64 repectively. 5407 symptoms were grouped into 26 SOCs. The most frequent SOCs in the 638 reports are nervous system disorders, general disorders, and administration site condition and investigations.

Analysis using zero-truncated Poisson model indicated that the avarage number of symptoms per subjects in the study cohort is 8.74 (95% CI 6.76, 10.73). There are statistically significant differences in number of symptoms among four age groups and between different genders. The youngest age group (0.5–17 years) has the smallest number of symptoms per year, followed by age group 2 (17–49), age group 4 (>64), and finally age group 3 (49–64). The average number of symptoms for subjects of 17–49 years old is 13% higher than the average number of symptoms for subjects of 0.5–17 years old with the same gender (p=0.003).

## Conclusion

This poster reports our preliminary analyses on influenza vaccine adverse events using VAERS data and MedDRA SOCs, which revealed differences of reactions among different age groups and between genders. The results may lead to additional studies to uncover factors contributing to the individual differences in susceptibility to influenza infection.
